# Non-anesthesiologist Administered Propofol Sedation in ASA III Patients: A Step too Far?

**DOI:** 10.1055/a-2893-8303

**Published:** 2026-06-22

**Authors:** Jianbo Wu

**Affiliations:** 1 Anesthesiology Department 66310 The First Affiliated Hospital of Shandong First Medical UniversityJinanShandongChina; 2 Shandong Institute of Anesthesia and Respiratory Critical MedicineJinanShandongChina; 3 Shandong Provincial Clinical Research Center for AnesthesiologyJinanShandongChina

## 

Propofol has revolutionized gastrointestinal endoscopy due to its rapid onset, reliable titration, and fast recovery, making it the preferred sedative for both endoscopists and patients. 1 Non-anesthesiologist-administered propofol (NAAP) refers to propofol sedation-targeted at moderate-to-deep sedation-delivered by non-anesthesiologist physicians. 2 Over the past decade, particularly in developed countries, NAAP has become standard practice for ASA I-II patients, with large registries and trials confirming its safety, efficiency, and cost-effectiveness when performed by properly trained providers. 3 Major guidelines, including the ESGE, endorse NAAP for average-risk patients. 4 5

However, rising global demand for endoscopy has driven a controversial expansion: NAAP use in ASA III patients with severe systemic diseases. Given their impaired physiological reserve, anesthesia societies have long discouraged NAAP in this high-risk group, recommending anesthesiologist involvement instead. 2 The 2010 ESGE guideline on NAAP was retracted in 2012 over safety concerns. 6 7 Subsequent guidelines from ASGE (2018), ESGE (2015), and the Chinese Stomatological Association (2022) all stress multidisciplinary collaboration, real-time monitoring, and immediate airway management capabilities for NAAP. 5 8 9 The 2026 Chinese Guidelines for Digestive Endoscopy Sedation further mandate repeated preoperative assessment and specialist consultation for ASA III patients. 10

In this issue of *Endoscopy International Open* , Bonura et al. present an 8-year retrospective study of NAAP in 1,423 ASA III patients undergoing diagnostic endoscopy. The overall anesthesia-related adverse event (ARAE) rate was 5.2%, with transient hypotension (3.6%) being the most common; no endotracheal intubation or sedation-related deaths occurred. The authors conclude NAAP is safe and feasible in ASA III patients when delivered by trained staff, potentially expanding endoscopic access without overloading anesthesiology services. This large single-center cohort provides valuable real-world data in an understudied high-risk population.

Other recent work supports non-anesthesiologist sedation in selected ASA III–IV patients, such as those undergoing TAVR, with low rates of emergency anesthetic support. 11

Nevertheless, broad adoption of NAAP in ASA III patients requires extreme caution. Propofol has a narrow therapeutic window and no reversal agent; even small dosing errors can trigger rapid cardiorespiratory compromise in patients with limited reserve. In the absence of outcome measures reported to at least 30 days after a procedure, can we be certain that ASA III patients exposed to procedural hypotension did not experience harm 12 ? Sometimes, to achieve more stable anesthesia and sedation, propofol may require adjunctive agents such as midazolam or ketamine, which may also lead to respiratory and circulatory complications. 13 The margin between moderate sedation and unplanned general anesthesia is dangerously narrow. Bonura et al. identified low BMI as the only independent risk factor for ARAEs, reflecting overestimated sedation tolerance in lean patients. The 2025 updated ASA classification designates BMI ≥ 40 kg/m ^2^ as ASA III, reinforcing the need for refined risk stratification.

Notably, obstructive sleep apnea (OSA), now classified as ASA III under updated criteria, further increases procedural risk. In ambulatory settings, OSA patients undergoing sedation carry a 1.7-fold higher odds of respiratory adverse events and a 3.3-fold higher odds of needing airway maneuvers. 14

Monitoring gaps also raise serious concerns. The Bonura study used pulse oximetry, ECG, and noninvasive blood pressure but omitted capnography. The ASA Closed Claims Database shows respiratory complications are nearly twice as common in nonoperating room anesthesia settings without mandatory capnography. 15 16 Supplemental oxygen can mask severe hypoventilation while maintaining normal SpO₂, meaning subclinical respiratory depression was likely underreported without capnography. 15 Recent data confirm capnography reduces hypoxia by nearly 40% during propofol sedation, and the 2026 Chinese Guidelines list it as mandatory for high-risk sedation. 10 17 Furthermore, the study’s excellent safety record relied on a dedicated sedation nurse-an ESGE requirement often neglected in resource-limited settings. 5

NAAP may be technically feasible in highly selected ASA III patients under controlled conditions with dedicated staff. However, feasibility must not be equated with generalizability. Routine NAAP in ASA III patients without mandatory capnography and standardized multicenter training remains premature.

To safely expand NAAP to higher-risk patients, future guidelines must require capnography as a standard of care. Combination sedation with midazolol and propofol should be strongly discouraged in ASA III patients. Until large prospective multicenter trials define safe ASA III subgroups, anesthesiologist involvement should be viewed as an essential safeguard, not a bureaucratic barrier. 17

The debate regarding whether sedation should be performed under the guidance of an endoscopist or an anesthesiologist will continue. 18 19 20 21 In European and American countries, non-anesthesiologist sedation is more widely adopted, whereas in Asian countries, anesthesiologist-led sedation predominates. Safety must remain the highest priority when balancing access, efficiency, and cost in endoscopic sedation. 2 21 In summary, Bonura et al. help define the risk boundaries of NAAP in ASA III patients. Yet their results depend on ideal monitoring and staffing. In the absence of mandatory capnography, standardized emergency protocols, and multicenter validation, routine NAAP in ASA III patients is still a step too far.

Future research should focus on precise risk stratification and multidisciplinary collaboration between anesthesiologists and endoscopists, ensuring that all patients receive safe and appropriate sedation regardless of ASA classification.
